# Herpes simplex virus type 2 in sub-Saharan Africa and the potential impact of helminth immune modulation

**DOI:** 10.3389/fcimb.2024.1471411

**Published:** 2024-12-04

**Authors:** Roxanne Pillay, Pragalathan Naidoo, Zilungile L. Mkhize-Kwitshana

**Affiliations:** ^1^ Department of Biomedical Sciences, Faculty of Natural Sciences, Mangosuthu University of Technology, Umlazi, South Africa; ^2^ Department of Medical Microbiology, College of Health Sciences, School of Laboratory Medicine & Medical Sciences, Nelson R. Mandela School of Medicine, University of KwaZulu-Natal, Durban, South Africa; ^3^ Division of Research Capacity Development, South African Medical Research Council (SAMRC), Tygerberg, Cape Town, South Africa; ^4^ Department of Biomedical Sciences, University of Johannesburg, Johannesburg, South Africa; ^5^ Biomedical Sciences Department of Life and Consumer Sciences, College of Agriculture and Environmental Sciences, University of South Africa, Johannesburg, South Africa

**Keywords:** sub-Saharan Africa, HSV-2, helminths, co-infection, HSV-2 outcomes, helminth immune modulation

## Abstract

Herpes simplex virus type 2 (HSV-2) and helminth infections are among the most widespread infectious diseases in sub-Saharan Africa (SSA). Helminths are known to modulate host immune responses and consequently impact the severity and outcomes of unrelated diseases, including allergies, autoimmune conditions, and infectious diseases. In this way, helminths may modulate essential immune responses against HSV-2 during co-infection and may alter susceptibility to and pathology of HSV-2. However, the epidemiology of STH/HSV-2 co-infections is understudied, and whether helminths influence the host immune response to HSV-2 is not well understood. In this perspective piece, we briefly examine the current knowledge on helminth immune modulation of important pathogens that are endemic to SSA, arguing that it is important to explore HSV-2 and helminth co-infections to elucidate potential interactions between HSV-2 and helminths. This is particularly relevant in SSA, where both pathogens are highly prevalent.

## 1 Introduction

Sub-Saharan Africa (SSA) bears a disproportionate and overlapping burden of Herpes simplex virus type 2 (HSV-2) (James et al., 2020) and helminth infections (Hotez and Kamath, 2009), therefore HSV-2 and helminth co-infections may likely occur. HSV-2, also known as genital herpes, is among the most common sexually transmitted viral infections (STVIs) (World Health Organisation, 2016). Helminths, including schistosomiasis and soil-transmitted helminthiasis, are common and widespread parasitic worm infections, particularly in SSA (Hotez and Kamath, 2009). Immunologically, helminth infections elicit potent T helper 2 (Th2) and immune modulatory responses, which dampen opposing T helper 1 (Th1) and T helper 17 (Th17) immune responses (Mcsorley and Maizels, 2012). In this way, helminth-induced immune modulatory effects are known to modulate host immune responses to unrelated pathogens, including important STVIs such as HIV (Mkhize-Kwitshana et al., 2011) and human papillomavirus (HPV) (Gravitt et al., 2015; Omondi et al., 2022). This in turn alters the pathology and clinical outcomes of important infections. In view of this, helminth immune modulation may potentially alter HSV-2 pathology and outcomes in individuals with HSV-2 and helminth co-infections. As effective host immunity to HSV-2 is primarily mediated by a Th1 response, helminth-induced Th2 and immune modulatory responses may hypothetically contribute to more severe outcomes of HSV-2. Nevertheless, there is a significant lack of evidence to validate this hypothesis. Moreover, despite their overlapping distribution in SSA, little is known about co-infections between helminths and HSV-2. Studying the epidemiological and immunological dynamics of these infections may help identify potential interactions and novel therapeutic interventions.

## 2 Method for literature search

A literature search was performed using search engines including Google Scholar, Google, PubMed, Web of Science and Science Direct to retrieve studies related to helminths and HSV-2 single and co-infections and their respective host immune responses. The search terms used included: “helminths”, “helminths and immune responses”, “HSV-2”, “HSV-2 and immune responses”, “helminths and HSV-2 co-infection”. In addition, to retrieve articles related to HSV-2 and/or helminths and their associations with infectious diseases endemic to SSA, the following search terms were used: “HSV-2 co-infections”, “HSV-2 and HIV”, “HSV-2 and HPV”, “HSV-2 and cervical cancer”, “HSV-2, HPV, and cervical cancer”, “helminth co-infections in Africa”, “helminths and malaria”, “helminths and TB”, “helminths and HIV”, “helminths and HPV”, “helminths and COVID-19”. The review focussed on the following article types, published in English: (i) review articles, (ii) human studies conducted in SSA, and (iii) experimental studies, where applicable. No year restrictions were applied.

## 3 Herpes simplex virus type 2 infections

HSV-2, which causes genital herpes, is a human DNA virus belonging to the Herpesviridae family and alpha subfamily (Chan et al., 2011). It is one of the most prevalent STVIs worldwide. An estimated 23.9 million incident HSV-2 infections were reported among individuals aged 15 – 49 years worldwide in 2016. Moreover, in 2016, the global prevalence was an estimated 13.2%, which equated to 491.5 million infections (James et al., 2020). There are significant differences in HSV-2 prevalence between continents, regions, and countries. Notably, the highest rates of infection are reported in countries with poor socio-economic conditions and under-resourced health facilities, particularly countries within SSA. HSV-2 seroprevalence in SSA is estimated at 33%, which is considerably higher compared to other global regions, such as estimated seroprevalences of 7% in Europe and 17% in the Americas (James et al., 2020). There are also considerable differences in HSV-2 prevalence between the subregions of SSA. Higher HSV-2 infection levels were recorded in Eastern Africa and Southern Africa, followed by Central Africa and Western Africa. Importantly, in SSA, nearly 50% of women and more than 25% of men were reportedly infected with HSV-2, demonstrating that women have a two-fold higher risk of infection (Harfouche et al., 2021).

HSV-2 transmission occurs via sexual contact with HSV-2-infected individuals during active viral shedding. The virus primarily targets the genital mucosa, replicating within keratinocytes of the genital epithelium. The natural progression of HSV-2 infection comprises three distinct phases: primary infection, latent infection, and reactivation. In immunocompetent individuals, most primary HSV-2 infections are self-limiting and asymptomatic, or may manifest as mild, non-specific symptoms (Schiffer and Corey, 2013). Symptomatic genital herpes is characterised by fever, body aches, lymphadenopathy, and dysuria, which resolve within 10 to 14 days. In addition, the classic feature of HSV-2 infection, occurring in 10 - 25% of initial infections, are painful genital vesicles or ulcers, which last approximately 3 weeks (World Health Organisation, 2016; Mathew Jr. and Sapra, 2024). Primary infection is followed by the latent phase, where the virus establishes latency in the sensory neurons and ganglia, leading to lifelong infection in humans. Cycles between latent and reactivated infection lead to recurrent symptoms, including genital lesions, genital ulcer disease, subclinical infections, and asymptomatic viral shedding (Chan et al., 2011). During reactivation, the virus travels along sensory nerves to the genital mucocutaneous site, replicates and forms herpetic lesions (Mathew Jr. and Sapra, 2024). Symptomatic recurrences typically occur within a year after initial HSV-2 infection and are less severe than the primary episode. In rare cases, systemic complications including recurrent meningitis, hepatitis and pneumonitis can occur during primary or reactivated infection, particularly in immunocompromised individuals due to AIDS, organ transplantation or chemotherapy. Although also rare, neonatal HSV infection occurs following viral transmission during childbirth and, when untreated, is associated with high mortality (>80%) and neurological morbidity (Schiffer and Corey, 2013; Mathew Jr. and Sapra, 2024).

Importantly, HSV-2 infection is characterised by intermittent viral shedding from the genital mucosa, in both symptomatic and asymptomatic individuals (World Health Organisation, 2016). Asymptomatic viral shedding from HSV-2-infected individuals contributes to the high prevalence of HSV-2. Diagnosis of HSV-2 infection is based on clinical presentation and laboratory detection of the HSV-2 virus, its viral proteins or genetic material, or HSV-2 specific antibodies. Several laboratory techniques may be used including: (i) viral culture from swab or needle aspirations; (ii) serology, such as enzyme-linked immunosorbent assays (ELISA) and Western blot assays to detect HSV-2 specific antigens and/or antibodies; (iii) and molecular-based methods, such as polymerase chain reaction (PCR) and loop-mediated isothermal amplification (LAMP) to detect the virus. Each of these methods has its benefits and drawbacks (Nath et al., 2021). However, these laboratory techniques require specialized equipment and personnel, and are therefore not accessible to a large proportion of populations, particularly in low- and middle-income countries (LMICs). LMICs face significant challenges with their healthcare systems and majority of their populations have limited access to healthcare facilities, leading to many undiagnosed HSV-2 infections. Moreover, because of its asymptomatic and/or non-specific clinical presentation, HSV-2 is often undiagnosed, or diagnosis and proper treatment may be delayed (Mathew Jr. and Sapra, 2024). Collectively, these factors exacerbate the spread of infection and increase the likelihood of co-infections with other prevalent pathogens.

Moreover, despite its high prevalence, there are currently no preventative or curative treatments for HSV-2 infection, and an effective vaccine is yet to be developed. Currently three approved classes of drugs are used to alleviate symptoms by targeting viral DNA replication and suppressing reactivation of HSV-2. These are acyclic guanosine analogues, acyclic nucleotide analogues, and pyrophosphate analogues. The common drugs from these classes include acyclovir, valacyclovir, cidofovir, and foscarnet. Acyclovir is the gold standard for treatment of HSV infections (Jiang et al., 2016). Unfortunately, because of its latent nature, HSV-2 causes lifelong infections and antivirals do not eliminate or prevent viral shedding. Concerningly, the emergence of antiviral drug resistance following long-term use and among immunocompromised patients has been reported, underscoring the urgent need to develop newer and effective therapeutic strategies (Jiang et al., 2016).

## 4 Immune responses during HSV-2 infection

Host immunity against HSV-2 involves components of the innate and adaptive immune responses that recognise, target, and lyse virally infected cells (Chan et al., 2011). The innate arm of immunity is critical as it forms the initial non-specific defence against HSV-2 infection and stimulates the adaptive immune response. The adaptive immune response plays an important role in viral clearance and development of long-term memory (Chan et al., 2011; Zhu et al., 2014).

The innate immune response is triggered by interaction between the virus and innate immune cells through pattern recognition receptors (PRRs), which detect pathogen-associated molecular patterns (PAMPs), such as viral DNA. The main PRRs, toll-like receptors (TLRs), occur on innate immune cells, including mononuclear phagocytes, dendritic cells (DCs), and natural killer (NK) T cells (Chew et al., 2009; Chan et al., 2011). Specific TLRs, such as TLR2 (Kurt-Jones et al., 2004), TLR3 (Zhang et al., 2007), TLR5 (Nazli et al., 2009), and TLR9 (Lund et al., 2006) have been shown to contribute to anti-HSV-2 innate immune responses. Following viral recognition and TLR activation, immune cells produce type I interferons (IFNs), thus stimulating an antiviral state through the activation of the RNA-dependent protein kinase (PKR) pathway via IFN-α1 transgene (Carr et al., 2005). Four main antigen-presenting cell (APC) subsets, Langerhans cells (LCs), CD14^−^ lamina propria (LP)-DCs, CD14^+^ LP-DCs, and macrophages, have been shown to regulate the antiviral state in the vaginal mucosa by polarizing CD4^+^ and/or CD8^+^ T cells through the expression of migration receptors (Duluc et al., 2013). Type I IFNs, mainly IFN-α and IFN-β, also promote an antiviral state by inhibiting translation and promoting the degradation of viral mRNA. In addition, type I IFNs support dendritic cell maturation and IL-15 production, which is needed for NK cell proliferation and survival. NK cells, in turn, secrete IFN-γ, and induce apoptosis of virally infected cells through the release of perforin and granzyme B. IFN-γ further enhances the anti-HSV-2 innate response by activating inducible nitric oxide synthase (iNOS). TLRs stimulate the production of proinflammatory cytokines, including IL-1, IL-6, and TNF-α, which contribute to the anti-HSV-2 innate immune response (Chan et al., 2011).

The adaptive immune response comprises cell-mediated and humoral responses. Adaptive immunity is triggered by the innate immune response, and promotes viral clearance and development of long-term memory (Zhu et al., 2014). During cell-mediated immunity, CD4^+^ T cells are recruited to the infection site and are activated by MHC class II antigen presentation on APCs. Activated CD4^+^ T cells release IFN-γ, stimulating epithelial cells to produce chemokines CXCL9 and CXCL10. A chemokine-driven gradient is created, which attracts cytotoxic CD8^+^ T cells to the infection site and stimulates nitric oxide (NO) release from epithelial cells and APCs. HSV-2-specific CD8^+^ T cells release IFN-γ and kill infected cells through perforin and fas-mediated pathways (Chan et al., 2011). Regulatory T cells (Tregs), which are known to suppress pathogen-associated immunity and tissue damage, have been found to play a role in HSV-2 infection. For example, mice depleted of Tregs, had reduced IFN levels in their draining lymph nodes (dLNs) and infection sites. In addition, there was delayed migration of NK cells, DCs, and T cells to infection sites, and increased proinflammatory chemokine levels in dLNs (Lund et al., 2008).

Lastly, during humoral responses, B cells are recruited to the infection site and are activated by CD4^+^ T cells to produce antibodies, IgA and IgG. However, the roles of B cells and antibodies remain debatable, given that HSV-2 viral glycoproteins have been shown to evade antibody-mediated protection [reviewed in (Chew et al., 2009; Chan et al., 2011; Zhu et al., 2014)].

## 5 Associations between HSV-2 and HIV, HPV, and cervical cancer

There are strong biological and epidemiological associations between HSV-2 and HIV; HSV-2 has been shown to be a key driver of the HIV epidemic in SSA, by biologically enhancing HIV acquisition and transmission by almost three-fold (James et al., 2020; Harfouche et al., 2021). HIV prevalence is highly prevalent in individuals co-infected with HSV-2 (Looker et al., 2017, 2020). Moreover, co-infection with HSV-2 has been shown to increase HIV viral shedding in genital secretions (Todd et al., 2013) and is associated with accelerated HIV disease progression (Lingappa et al., 2010; Reynolds et al., 2012). The underlying biological mechanisms by which HSV-2 increases the risk of HIV infection, include a compromised genital epithelium barrier, an influx and increased number of target cells in genital tissue for HIV entry, decreased innate mucosal immunity, and chronic mucosal inflammation (Zhu et al., 2014).

Several studies have explored the potential association between HSV-2 and human papillomavirus (HPV), particularly in terms of co-infection (Francis et al., 2018; Taku et al., 2021; Uysal et al., 2022) and the potential risk for cervical cancer development (Smith et al., 2002; Zhao et al., 2012; Martin Luther et al., 2014; Li and Wen, 2017; Yousif Elemam et al., 2018; Zhang et al., 2023).

Studies have shown that HSV-2-infected individuals are more likely to have concurrent HPV infections; co-infection is reported more frequently in regions with high prevalences of both viruses, and particularly among women (Francis et al., 2018; Taku et al., 2021; Uysal et al., 2022). HSV-2 infection has been associated with an increased risk of acquiring HPV. Biologically, inflammation and disruption of genital epithelial barriers caused by HSV-2 genital ulcers, can facilitate the transmission of other viruses, including HPV (Sausen et al., 2023).

It is well known that persistent infection with high-risk strains of HPV, mainly HPV-16 and HPV-18, is the primary cause of cervical cancer (De Sanjosé et al., 2018). What is less understood, however, is the role of HSV-2 in cervical carcinogenesis. In 2020, cervical cancer affected an estimated 604,000 women and accounted for 342,000 deaths globally. SSA has the highest cervical cancer incident and mortality rates, and cervical cancer is the leading cause of cancer-related deaths among women in SSA (Sung et al., 2021). This highlights that despite the availability of an effective vaccine against HPV, cervical cancer is still a significant public health concern, particularly in SSA. Moreover, this suggests that several other factors may contribute to cervical cancer pathogenesis.

Whether HSV-2 infection alone, or in conjunction with HPV, impacts the development of cervical cancer remains debatable, with studies yielding conflicting results (Sausen et al., 2023). However, there is some evidence to suggest that co-infection with HSV-2 may increase the risk of HPV-related cervical cancer (Smith et al., 2002; Zhao et al., 2012; Martin Luther et al., 2014; Li and Wen, 2017; Yousif Elemam et al., 2018; Zhang et al., 2023). Several mechanisms by which HSV-2 may contribute to cervical cancer development have been described: (i) HSV-2-associated genital ulcers may facilitate HPV entry to the basal layer; (ii) HSV-2 induces an inflammatory response, which may impair T helper cell mediated immune responses; (iii) HSV-2 acts directly on host cellular DNA, and induces the production of nitric oxide, resulting in cellular DNA damage; (iv) HSV-2 infection accelerates replication of HPV and its integration of viral DNA sequences; (iv) both HSV-2 or HPV infections may induce immunological and microbiological changes, such as dysbiosis of the vaginal microbiota. Such changes could potentially create a conducive environment for HPV persistence, HSV-2 reactivations, and progression to cervical cancer (Al-Daraji and Smith, 2009; Uysal et al., 2022; Sausen et al., 2023). Thus, establishing the role of HSV in cervical carcinogenesis may have important implications for mitigating the occurrence of cervical cancer. Should HSV-2 contribute to cervical cancer, then its timely diagnosis and treatment could become a potential therapeutic avenue to curb the disease.

Apart from the significant burdens of HSV-2, HIV, HPV, and cervical cancer in SSA, the continent bears a substantial burden of other infectious diseases, such as tuberculosis (TB), malaria, as well as neglected tropical diseases (NTDs), such as helminths (Hotez and Kamath, 2009). Given this geographic overlap of multiple infectious diseases, the occurrence of concurrent infections is highly likely, with profound consequences for individual and public health in SSA. Herein, we hypothesise the potential impact of helminth immune modulation on HSV-2 pathology in SSA.

## 6 Helminth infections

Helminths are endemic to SSA and contribute to approximately 85% of the continent’s NTD burden. Helminths are associated with extreme poverty, causing chronic and insidious infections that negatively impact child development, pregnancy outcomes and economic productivity (Hotez and Kamath, 2009). Most infections are caused by the four major soil transmitted helminths (STHs) (*Ascaris lumbricoides*, *Trichuris trichiura*, *Necator americanus* and *Ancylostoma duodenale*) (World Health Organisation, 2024b). According to the World Health Organisation (WHO), approximately 232 million pre-school and school-aged children residing in the WHO African region are at risk of infection (World Health Organisation, 2024c). Other vulnerable groups include women of reproductive age, and adults working on tea farms and in mines (World Health Organisation, 2024b).

STHs are transmitted via faecal contamination of food and environmental sources. Individuals become infected with *Ascaris lumbricoides* and *Trichuris trichiura* infections when they ingest embryonated eggs found in contaminated water or food, and with hookworms, when infective larvae penetrate the skin. STH have complex lifecycles, sometimes requiring multiple hosts to successfully complete their developmental stages, which comprise larval migration through one or more host tissues, maturation into adult worms, reproduction, and the excretion of new eggs into the environment (Bethony et al., 2006). STHs require similar diagnostic methods and respond to the same treatment. Large-scale efforts to reduce STH-associated morbidity in at-risk groups residing in endemic regions, include STH preventative chemotherapy using the benzimidazole anthelmintics, albendazole (400 mg) and mebendazole (500 mg) (World Health Organisation, 2024b).

Human schistosomiasis is a parasitic disease caused by trematodes of the genus *Schistosoma.* There are two major forms of disease, intestinal and urogenital schistosomiasis. Three main species of schistosomes infect human beings, *Schistosoma mansoni* and *Schistosoma japonicum* (intestinal schistosomiasis), and *Schistosoma haematobium* (urogenital schistosomiasis) (Colley et al., 2014). It is estimated that at least 264 million people worldwide are infected with *Schistosoma* spp (World Health Organisation, 2024a). Schistosomiasis is associated with significant morbidity and mortality. More than 90% of the infections occur in Africa, an estimated two thirds of infections are caused by *Schistosoma haematobium* (Santos et al., 2021).

Schistosomiasis transmission occurs when infected individuals contaminate freshwater sources with faeces or urine containing parasite eggs, which then hatch into larvae (cercariae) in the water. Infection occurs when larvae, released by aquatic snails, penetrate the skin during contact with contaminated water. Within the human host, larvae mature into adult schistosomes and migrate through the blood vessels. Adult schistosomes pair up and colonise the blood vessels for many years, where they produce eggs. Some of these eggs are excreted through faeces or urine, continuing the parasite’s lifecycle, while others become lodged in the intestines or liver (*Schistosoma mansoni* and *Schistosoma japonicum)*, or walls of the urinary tract and bladder (*Schistosoma haematobium)* (Odegaard and Hsieh, 2014; Colley et al., 2014). Moreover, the embedded eggs induce a chronic, distinct immune-mediated granulomatous response that has local and systemic pathological consequences (Colley et al., 2014).

Standard diagnostic methods for schistosomiasis include detection of viable eggs in urine or stool samples, or tissue biopsies, using techniques such as microscopy and the Kato-Katz method. However, these methods suffer from low sensitivity, and the true burden of schistosomiasis may be underestimated (Colley et al., 2014). Schistosomiasis control focuses on periodic, large-scale treatment of at-risk populations with praziquantel, to reduce morbidity. While praziquantel is safe to administer and effective against adult schistosomes, poor efficacy against immature schistosome larvae is reported (Colley et al., 2014).

## 7 Immune responses during helminth infections

Helminths and their human hosts have co-evolved over many centuries; thereby helminth parasites have developed several mechanisms to ensure their longevity in infected hosts. Typically, helminth-induced tissue injury stimulates a Th2 host immune response, which supports wound repair and reduces tissue inflammation caused by helminths as they migrate through different host tissues and organs. Initial helminth-induced tissue injury activates the innate immune response, where the release of danger associated molecular patterns (DAMPS) stimulate epithelial cells to release alarmin cytokines [IL-25, IL-33, and thymic stromal lymphopoietin (TSLP)]. IL-25 and IL-33 stimulate innate cells to produce Th2-associated cytokines (IL-4, IL-5, and IL-13), while TSLP suppresses the production of IL-12, a major Th1 cytokine, and supports dendritic cell maturation (Harris and Loke, 2017; Rapin and Harris, 2018). The host adaptive immune response to helminth infection is important for stimulating the development of Th2 cells and their associated cytokines, helminth expulsion, and preventing re-infection (Harris and Loke, 2017). Helminth-induced Th2 responses can downregulate Th1 and Th17 immune responses and their associated cytokines, such as IL-12, IFN-γ, IL-17, IL-23, and TNF-α. Moreover, helminths can dampen Th1 and Th2 host immune responses through the expansion of regulatory cell populations (FOXP3^+^ T regulatory and B regulatory cells) and alternatively activated macrophages (AAMs). Collectively, these regulatory cell populations stimulate the release of immunosuppressive cytokines, IL-10, and tumour growth factor (TGF-β), thus creating a hyporesponsive or tolerant environment in the infected host that promotes helminth survival and limits host tissue damage (Mcsorley and Maizels, 2012).

## 8 Bystander effects of helminth immune modulation on important pathogens

The immune modulatory effects of helminths can influence a range of unrelated bystander conditions and infections. The influence of helminth immune modulation on inflammatory conditions, such as allergies and autoimmune diseases, has been described. According to the hygiene hypothesis, the absence of helminths in developed regions, due to improved sanitation and hygiene, has led to an increase in allergic conditions and autoimmune diseases, compared to helminth-endemic regions (Maizels and Mcsorley, 2016). In support, evidence emerging from Africa, demonstrate decreased prevalence of atopy (Van Den Biggelaar et al., 2000) and decreased levels of autoreactive antibodies in helminth-infected individuals (Mutapi et al., 2011).

Helminth immune modulation has been shown to influence susceptibility to and the clinical course of infectious diseases that are endemic to SSA, with varying outcomes. Some key examples from studies conducted in SSA are provided here.

Studies of helminth-malaria co-infection have yielded conflicting results. Helminth co-infection was associated with a higher prevalence of non-severe malaria (*Plasmodium falciparum* or *Plasmodium vivax*) (Degarege et al., 2012; Babamale et al., 2018), and with a higher intensity of *Plasmodium falciparum* (Babamale et al., 2018). Moreover, individuals co-infected with STHs and *Plasmodium falciparum* had more pronounced malnutrition, anaemia and lower body weight, suggesting that co-infection with STHs exacerbates malnutrition, anaemia, low body weight status (Degarege et al., 2010). STHs are associated with anaemia and nutritional deficiencies (Stephenson et al., 2000; Mpaka-Mbatha et al., 2022). STHs may suppress appetite due to gut inflammatory responses mediated by the infected host. Some STHs (*Ascaris lumbricoides* and hookworms) secrete inhibitors of pancreatic enzymes, which may directly impair host nutrient absorption in the small intestine (Cappello, 2004). Hookworms colonise the small intestine, where they feed on blood. High intensity hookworm infections are associated with iron deficiency anaemia, particularly in children and women of reproductive age, who are malnourished or have low iron levels (Loukas et al., 2021).

Helminth immune modulation has also been shown to influence *Mycobacterium tuberculosis.* A strong association between helminth infection and active TB was reported in a cohort of Ethiopian patients (Elias et al., 2006), suggesting that helminth immune modulation impairs host immune responses to TB. Moreover, in helminth-infected individuals, purified protein derivative (PPD) and T cell responses were impaired in response to natural immunisation and bacille Calmette-Guérin (BCG) vaccination, respectively, but improved following anthelmintic treatment (Elias et al., 2001). Chronic helminth infection was associated with reduced BCG efficacy and correlated with increased levels of TGF-β (Elias et al., 2008).

Sexually transmitted viral infections are highly endemic to SSA. Given the significant burden of HIV in SSA, many studies have focussed on helminth-HIV interactions. In this context, a high prevalence of helminths was reported among HIV-infected individuals (Mkhize-Kwitshana et al., 2011; Ivan et al., 2013; Abossie and Petros, 2015). In helminth/HIV co-infected individuals, CD4^+^ counts were lower (Adeleke et al., 2015), immune cells were dysregulated, and HIV viral loads were higher (Mkhize-Kwitshana et al., 2011). In addition, hookworm infection was correlated with a higher risk of HPV infection, higher HPV viral loads, and distinct mixed Type 1/Type 2 immune profiles in the vaginal tracts of helminth/HPV co-infected women (Gravitt et al., 2015; Holali Ameyapoh et al., 2021; Omondi et al., 2022).

*Schistosoma haematobium* infection, which causes urogenital schistosomiasis, can profoundly impact reproductive health. In chronically infected women, vaginal pathology is associated with itching, bleeding, pain, discharge, genital lesions, genital tumours, and ectopic pregnancies (Hegertun et al., 2013; Norseth et al., 2014). In men, urogenital schistosomiasis is associated with pathology of the seminal vesicles, dysuria, haematuria, pelvic pain, and infertility (Kayuni et al., 2019; Roure et al., 2024). Importantly, *Schistosoma haematobium* is classified as a group 1 carcinogen; the correlation between urogenital schistosomiasis and bladder cancer has been previously described (Salem et al., 2011; Khaled, 2013; Ishida and Hsieh, 2018). Urogenital schistosomiasis is also associated with an increased risk of HIV infection (Looker et al., 2017), particularly in women (Kjetland et al., 2006; Ndhlovu et al., 2007; Downs et al., 2011). Biologically, chronic inflammation and tissue damage caused by *Schistosoma haematobium* eggs, enhanced immune activation, and genital lesions in the female reproductive tract, are thought to increase the risk of HIV, by increasing viral entry points and the number of target cells at the infection site. Additionally, *Schistosoma haematobium*-associated Th2 immune response may suppress the Th1 responses needed for effective anti-HIV immunity (Chetty et al., 2020).

When holistically evaluating the effects of helminth immune modulation, it is important to mention that helminths may have beneficial effects on concurrent infections. This was reported during the recent COVID-19 pandemic; helminth co-infection (*Hymenolepis nana, Schistosoma mansoni* and *Trichuris trichiura*) mitigated COVID-19 severity in patients from Africa (Wolday et al., 2021).

Taken together, it is evident that different factors determine whether helminths have beneficial or detrimental effects on concurrent bystander infections. These include the helminth species, the worm burden, type of concurrent infection/condition, tissue tropism, and the timing and niche of infection (Schlosser-Brandenburg et al., 2023).

## 9 Discussion: potential implications of helminth immune modulation on HSV-2

**Figure 1:**
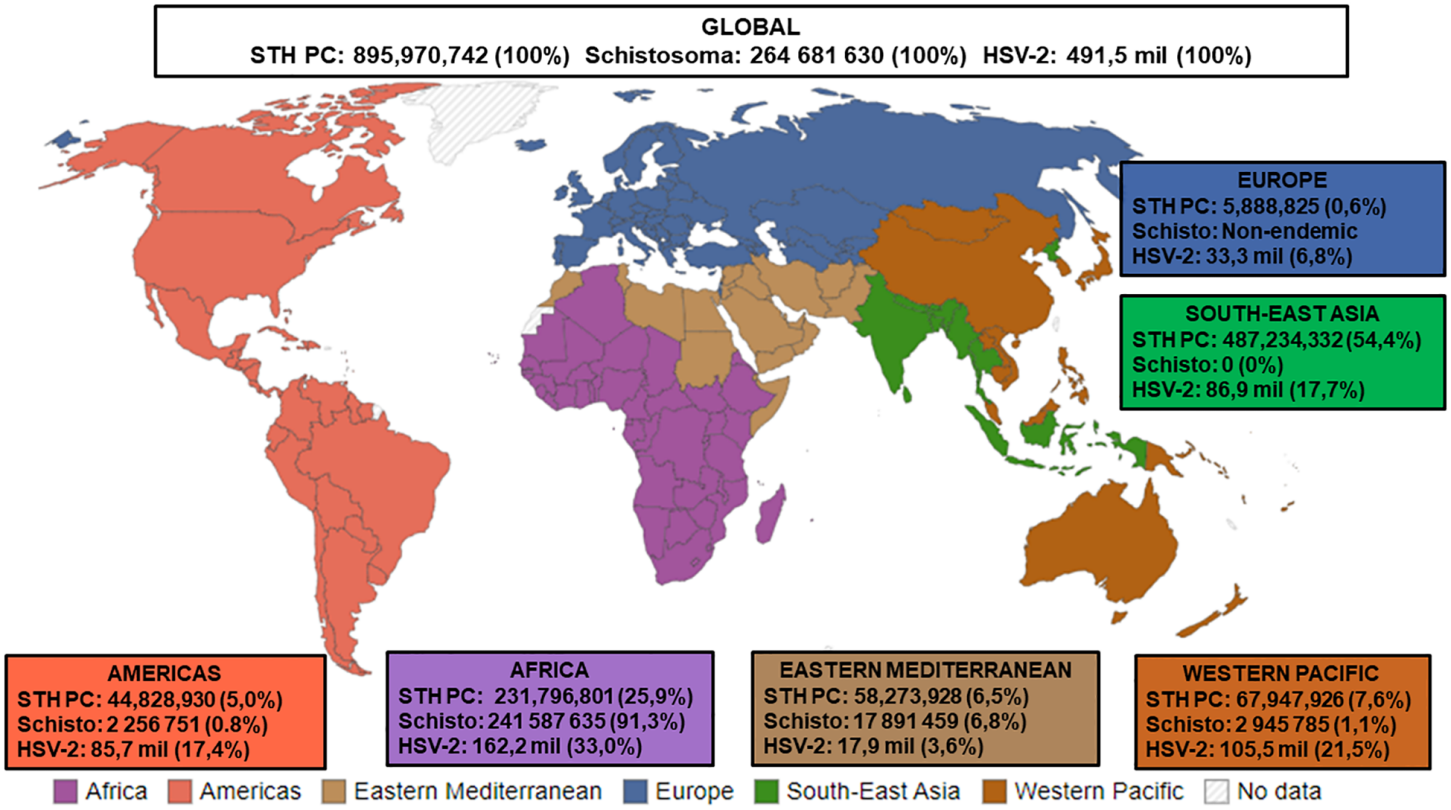
Estimated numbers (n) and proportions (%) of STH PC required for pre-SAC and SAC, Status of Schistosomiasis in endemic countries, and HSV-2 infections. STH PC: Soil-transmitted helminths Preventive Chemotherapy [Data presented is the estimated number of Pre-school Aged (Pre-SAC) and School-Aged Children (SAC) requiring PC for STHs in 2022^a^; % Proportion = Estimated number of STH PC/Estimated number of Global STH PC x 100]; Schisto: Status of Schistosomiasis in endemic countries in 2022^b^; % Proportion = Estimated number of Schistosoma/Estimated number of Global Schistosoma PC x 100]; HSV-2: Herpes Simplex Virus Type II [Data presented is the estimated number of people within the 15-49 year age group that were infected with HSV-2 in 2016^c^; % Proportion = Estimated number of HSV-2/Estimated number of Global HSV-2 x 100, Source of WHO regions map^d^. ^a^Source: Adapted from (World Health Organisation, 2024c). ^b^Source: Adapted from (World Health Organisation, 2024a). ^c^Source: Adapted from (James et al., 2020). ^d^Source: Adapted from (Our World in Data, 2023).

Considering the studies described above, it is plausible that helminth immune modulation may influence HSV-2 outcomes in SSA. Given the substantial prevalence and geographic overlap of HSV-2 and helminths in SSA, it is likely that co-infections occur (**Figure 1**). However, no studies have examined the influence of helminth immune modulation on HSV-2 co-infection in humans. However, in a very recent murine study, Chetty et al. (2021) demonstrated that acute, self-limiting infection with *Nippostrongylus brasiliensis*, a murine intestinal helminth that is closely related to the human hookworms, induced a classic Th2 immune response in the female genital tract (FGT). FGT immune responses to subsequent genital HSV-2 were impaired, leading to enhanced HSV-2 pathology. This enhanced pathology was dependent on IL-5 and associated with increased levels of IL-33, group 2 innate lymphoid cells (ILC2s), and accumulation of eosinophils in the FGT (Chetty et al., 2021). The exact mechanisms by which *Nippostrongylus brasiliensis* influences the FGT, a site it does not colonise, need to be further explored. It has been suggested that helminth-derived excretory-secretory products may play a key role in inducing immune cell trafficking and Th2 responses in uncolonized tissues, such as the FGT (Zarek and Reese, 2021). Importantly, Chetty et al. (2021) demonstrate the systemic effects of helminth infection on FGT immune responses to HSV-2 infection, which supports previously observed clinical correlations between STHs and viral infections in vaginal tissue (Gravitt et al., 2015; Omondi et al., 2022). Thus, their novel findings provide some insight into how helminths may alter HSV-2 pathology in co-infected individuals and provides a basis for future human studies. Given that helminths stimulate Th2 and immune modulatory responses, whereas protection from HSV-2 requires a Th1 response, we hypothesise that helminths may compromise host immunity to HSV-2 during co-infection. This could enhance susceptibility to HSV-2, promote viral persistence and pathology, and impair responses to HSV-2 treatment. Therefore, helminth-HSV-2 co-infections could have significant consequences for sexual and reproductive health, such as increased risk of acquiring other sexually transmitted infections, infertility, and cancer progression (Chetty et al., 2020). The ensuing negative impact on inadequately resourced healthcare systems in SSA would be substantial. Furthermore, it may be necessary to consider how helminth immune modulation may impact HSV-2 treatment efficacy; this factor may need to be considered when designing appropriate treatment strategies and potential HSV-2 vaccines. We argue that helminth/HSV-2 co-infections are grossly understudied, therefore their potential impact on public health is underestimated. To address this gap, we believe that human studies exploring helminth/HSV-2 co-infections in SSA are warranted. Moreover, we believe that evaluating and managing these infections using holistic and integrated approaches may advance the WHO 2030 Sustainable Development Goals aimed at eliminating NTDs and sexually transmitted infections (World Health Organisation, 2022a, 2022).

## 10 Concluding remarks

HSV-2 and helminth co-infections may commonly occur in SSA. However, their potential interactions are grossly understudied and poorly understood. There is a significant paucity of data on HSV-2 and helminth co-infections, and whether helminth immune modulation influences HSV-2 pathology is unclear. We assert that epidemiological and immunological studies on HSV-2 and helminth co-infections are needed to fully understand the interplay between these pathogens. Moreover, evidence from such studies could prove relevant as they may inform the therapeutic management of HSV-2 and helminths in co-endemic regions such as SSA.

## Data Availability

The original contributions presented in the study are included in the article/supplementary material. Further inquiries can be directed to the corresponding author.
